# Electrocardiogram Cream Reduces Skin-Electrode Impedance Upon Neuromuscular Monitoring Using TOF-Cuff®

**DOI:** 10.7759/cureus.44670

**Published:** 2023-09-04

**Authors:** Sho Sugimura, Huynh V Khanh, Shingo Kawashima, Yoshiki Nakajima, Hiroyuki Kinoshita

**Affiliations:** 1 Departments of Anesthesiology and Intensive Care, Hamamatsu University School of Medicine, Hamamatsu, JPN; 2 Departments of Anesthesiology and Intensive Care Medicine, Hamamatsu University School of Medicine, Hamamatsu, JPN; 3 Department of Dental Anesthesiology, Tokushima University Graduate School of Biomedical Sciences, Tokushima, JPN

**Keywords:** muscle relaxant, neuromuscular block monitoring, neuromuscular monitoring, impedance, electrocardiogram cream

## Abstract

Background

Mechanistic insight into the high failure rate of TOF-Cuff® (RGB Medical Devices, Madrid, Spain) measurements on the lower leg is unclear.

Aims

We aimed to determine whether materials applied to pseudo-skin can reduce the impedance between a model arm and TOF-Cuff® electrodes and whether a material between TOF-Cuff® electrodes and the patient’s skin surface decreases the skin-TOF-Cuff® electrode impedance within the appropriate range.

Methods

This was a combination of an in vitro study using non-living materials and a prospective observational clinical study. Eight patients aged > 70 years who had undergone elective surgery were eligible. One of the primary outcomes was whether water, electrocardiogram (ECG) cream, or ECG gel applied on the pseudo-skin could reduce the impedance between the model arm and the TOF-Cuff® electrodes in the in vitro study. Another was whether a material between the TOF-Cuff® electrodes and the patient’s skin surface decreased the skin-TOF-Cuff® electrode impedance to an appropriate level of less than 5,000 Ω in the clinical study.

Results

The application of water, ECG cream, and ECG gel similarly reduced the impedance values within the electrical circuit in the in vitro study. ECG cream application between the patient’s skin surface and the TOF-Cuff® electrodes decreased the skin-TOF-Cuff® electrode impedance (median (interquartile range (IQR)) Ω) from 8,600 (6,450 to 9,775) to 2,000 (1,600 to 2,600) (P = 0.012) in surgical patients.

Conclusion

ECG cream application between the patient’s skin surface and the TOF-Cuff® electrodes decreased the skin-TOF-Cuff® electrode impedance appropriately, and thus, the application can facilitate precise TOF-Cuff® measurements in patients.

## Introduction

There is evidence that quantitative neuromuscular monitoring reduces the risk of residual neuromuscular blockage [1]. The TOF-Cuff® (RGB Medical Devices, Madrid, Spain), a myograph monitoring device consisting of a modified blood pressure cuff with two electrodes on the inside, is targeted for neuromuscular monitoring during surgical procedures where a free-moving thumb is interrupted [2-4]. TOF-Cuff® determines a neuromuscular block status using the pressure changes generated by muscular activity in the inner part of the cuff following peripheral nerve stimulation at the upper arm or the lower leg [5]. Neuromuscular monitoring in the lower leg could be an alternative when both hands and arms are unavailable during surgery [6,7]. However, a recent study reported a high failure rate with TOF-Cuff® measurements on the lower leg, although mechanistic insight into the failure has been unclear [8].

The technical artifacts of electromyography monitoring include monitoring cable motion, transducer noise caused by electrode displacement, high skin-electrode impedance, intrinsic noise within the electromyograph monitor, and nearby biomedical devices such as a pacemaker [9]. Of these factors, we thought that one might be capable of appropriately handling the skin-electrode impedance by adding some modification to the patient’s skin surface during TOF-Cuff® monitoring. However, specific procedures that reduce the patient’s skin-TOF-Cuff® electrode impedance are necessary because an electrode for electromyography monitoring must be implemented with a low impedance, usually less than 5,000 Ω [9].

Therefore, we conducted the following two studies to decrease impedance during TOF-Cuff® use in clinical practice. We first examined the materials applied to pseudo-skin between a model arm and the TOF-Cuff® electrodes to reduce the impedance within the electrical circuit in the in vitro study. In addition, using one of the materials applied between the patient’s skin surface and the TOF-Cuff® electrodes decreases the skin-TOF-Cuff® electrode impedance to an appropriate level of less than 5,000 Ω in the clinical study.

## Materials and methods

The current study contained two phases, including the in vitro study, in which the materials applied between pseudo-skin and TOF-Cuff® electrodes can reduce electrical resistance, and the prospective, observational clinical study, in which the application of a particular material between a patient’s skin surface and TOF-Cuff® electrodes decreases the skin-electrode impedance. The studies were performed in March 2023 at the Departments of Anesthesiology and Intensive Care, Hamamatsu Medical University, Hamamatsu, Japan. Ethical approval for this study (IRB approval number: 22-159) was provided by the Clinical Research Review Board of the Hamamatsu University School of Medicine, Hamamatsu, Shizuoka, Japan, on January 26, 2021. This study was registered in the University Hospital Medical Information Network Clinical Trials Registry (UMIN000051409). Written informed consent was obtained from all patients prior to their enrolment in the clinical study. The procedures in the current study followed the “Declaration of Helsinki” and the ethical standards of the responsible committee on human experimentation. Patients >70 years old who underwent general anesthesia were included in the clinical study. Patients with obesity, emaciation, or evident skin disease at the measurement site were excluded.

A previous study documented that an electrode for electromyography monitoring must be implemented with low electrical resistance, usually less than 5,000 Ω [9]. Therefore, the primary outcomes of the current study are as follows. One was whether water, electrocardiogram (ECG) cream, or ECG gel applied on pseudo-skin between a model arm and TOF-Cuff® electrodes could reduce the impedance within the electrical circuit to less than 5,000 Ω in the in vitro study. Another was whether applying a material between the patient’s skin surface and the TOF-Cuff® electrodes decreases the skin-TOF-Cuff® electrode impedance to an appropriate level of less than 5,000 Ω in the clinical study.

In vitro impedance measurement using a model arm

**Figure 1:**
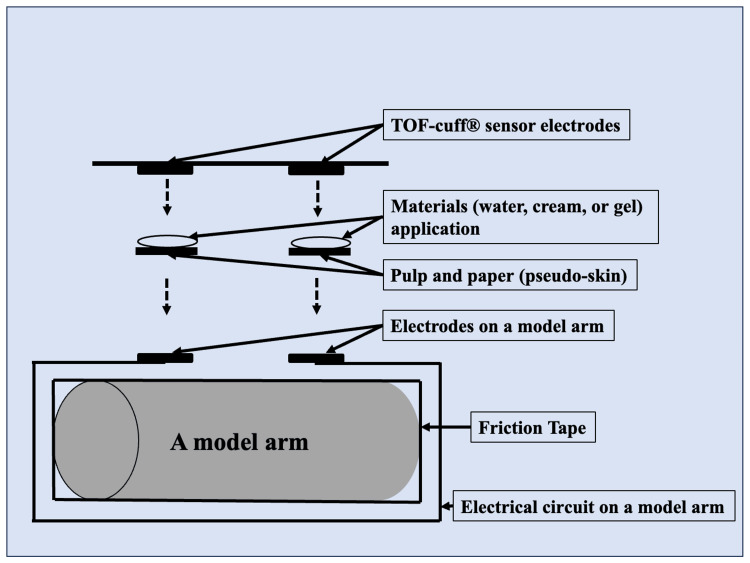
The structure of the model arm in the current study The structure of the model arm with pulp and paper for the impedance measurement in the current study is shown. Both ends of the electrical circuit are attached to the electrodes on the model arm. Pulp and paper (a pseudo-skin) with and without material, including water, electrocardiogram (ECG) cream, or ECG gel, existed between TOF-Cuff® sensor electrodes and the electrodes on the model arm.

**Figure 2:**
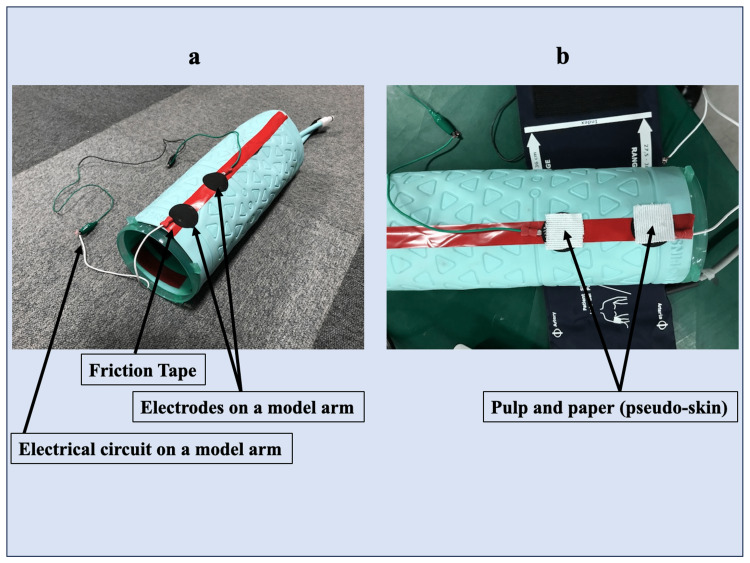
The actual setting of a model arm The actual setting of a model arm with and without pulp and paper for the impedance measurement. a. A model arm setting with two electrodes, a friction tape, and an electrical circuit. b. A model arm setting with two pieces of white pulp and paper on the top of the model arm’s electrodes.

TOF-Cuff® equips a unique “service mode” for automatic impedance measurement between its electrodes to meet the demand. We used this system in the current in vitro study. First, we prepared a model arm made from polyvinyl chloride, covered by friction tape and an electrical circuit (Figures 1, 2a). Then, we placed two electrodes with the exact distance between them as the TOF-Cuff® sensor electrodes (Figure 1). In this in vitro study, two pieces of pulp and paper were used as pseudo-skin between the electrodes on a model arm and the TOF-Cuff® sensor electrodes for impedance measurement (Figures 1, 2a, 2b). Finally, we examined the effects of materials including water, electrocardiogram (ECG) cream (KENZ ECG Cream^TM^, SUZUKEN Inc., Nagoya, Aichi, Japan), and ECG gel (Smart Veil^TM^, SHALOM Inc., Minamitsuru-gun, Yamanashi, Japan) on the pulp and paper (pseudo-skin) surface application on the side of the TOF-Cuff® sensor electrodes (Figure 1).

Both electrodes were cleaned on a model arm and TOF-Cuff® sensor before each impedance measurement using 70% ethanol and dried. Then, we placed the TOF-Cuff® sensor around a model arm without pulp and paper as the model arm electrodes, and the electrodes of the TOF-Cuff® sensors faced precisely. First, we measured the impedance without pulp and paper (pseudo-skin) between the electrodes (Figure 2a). Second, we placed pulp and paper (pseudo-skin) between the model arm electrodes and the TOF-Cuff® sensor electrodes and evaluated the impedance values similarly (Figure 2b). Finally, we applied water, ECG cream, or ECG gel to the pulp and paper (pseudo-skin) surface on the side of the TOF-Cuff® sensor electrodes and measured the impedance value. We repeated the above protocol 10 times in a series of in vitro studies. Each measurement was completed within five minutes.

Clinical impedance evaluation in patients undergoing general anesthesia

None of the patients had received premedication. General anesthesia for each patient under 100% face mask oxygen (6 L/min) was induced with 1 mg/kg IV propofol. Bag-mask ventilation was continued under 2% sevoflurane inhalation in 6 L/min oxygen combined with 0.05 µg/kg/min of IV remifentanil, maintaining endo-tidal carbon dioxide tension between 35 and 40 mmHg.

We implemented a single train-of-four measurement for each patient using TOF-Cuff® on either ankle, as the sensor was attached to the posterior tibial nerve. The set of train-of-four measurements using TOF-Cuff® was performed with and without ECG cream application on the patient’s ankle skin. Whether a technical alarm showing “high patient impedance,” which indicates the impedance between the patient’s skin and TOF-Cuff® electrodes above 8,000 Ω (the TOF-Cuff® manual provided by RGB Medical Devices, and translated into Japanese by IMI Co., Ltd., Koshigaya, Saitama, Japan), was displayed on the TOF-Cuff® monitor was examined in the clinical study. Thereafter, we extracted impedance data on the train-of-four measure using the “export trends data option” in the TOF-Cuff® monitor.

Statistical analysis

Statistical analyses were performed using the IBM SPSS^TM^ Statistics version 27 (IBM Japan Inc., Tokyo, Japan). Data are shown as the median and interquartile range (IQR) for continuous variables, while Wilcoxon signed-rank tests were performed for group comparisons. Differences were considered statistically significant when P was < 0.05.

The power calculation for the clinical study was performed using G*power Version 3.1.9.6 (Heinrich-Heine-Universität Düsseldorf, Düsseldorf, Germany). Assuming that a 1,500 Ω difference in impedance is clinically significant, we calculated that six patients would need to have 80% power at a two-sided α level of 0.05 to show the impedance level difference of 1,500 Ω. Enrolled patients increased by eight in the current study to allow for potential unevaluable patients and incomplete patient data collection. Indeed, a 6,600 Ω difference in the impedance measure gave 99% power to detect a significance level of α = 0.05, with a sample size of four (SD = 1664) in the current study.

## Results

**Figure 3:**
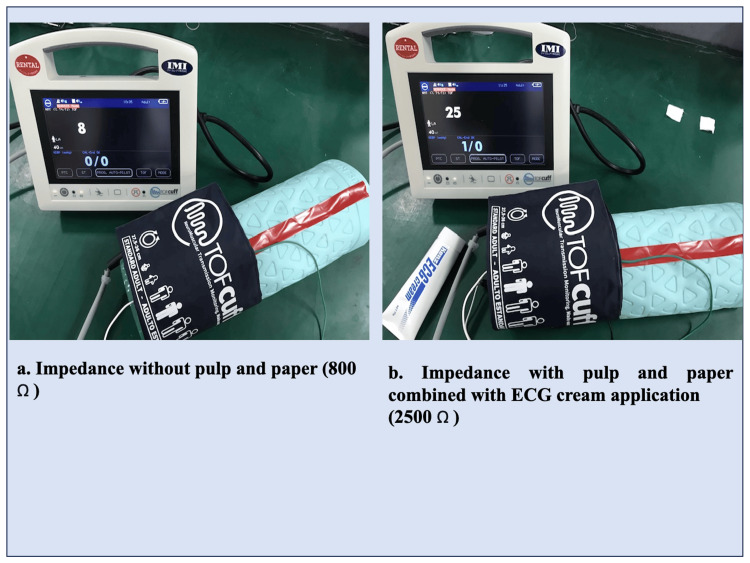
Impedance measurement using the model arm a. A representative impedance measurement without pulp and paper using the model arm in the current study. Please note that the TOF-Cuff® display demonstrates 8, showing 800 Ω. b. A representative impedance measurement with pulp and paper combined with electrocardiogram (ECG) cream application, documenting 2,500 Ω.

A representative impedance measurement using the model arm without pulp and paper and with pulp and paper combined with ECG cream application is shown in Figure 3. Water, ECG cream, and ECG gel application similarly reduced the impedance values between the model arm and TOF-Cuff® electrodes in the in vitro study (Table 1).

**Table 1:** In vitro impedance measurement using a model arm Impedance measurements (Ω), in which pulp and paper are placed on the TOF-Cuff® electrodes in combination with and without water, the ECG cream, or the ECG gel (n = 10 each). Data are shown as median (interquartile range). ECG: electrocardiogram.

	Without each application	With each application	P
Water	11,800 (11,800 to 11,800)	2,000 (2,000 to 2,100)	0.004*
ECG cream	11,800 (11,800 to 11,800)	2,500 (2,500 to 2,725)	0.005*
ECG gel	11,800 (11,800 to 11,800)	2,050 (2,000 to 2,200)	0.004*

**Figure 4:**
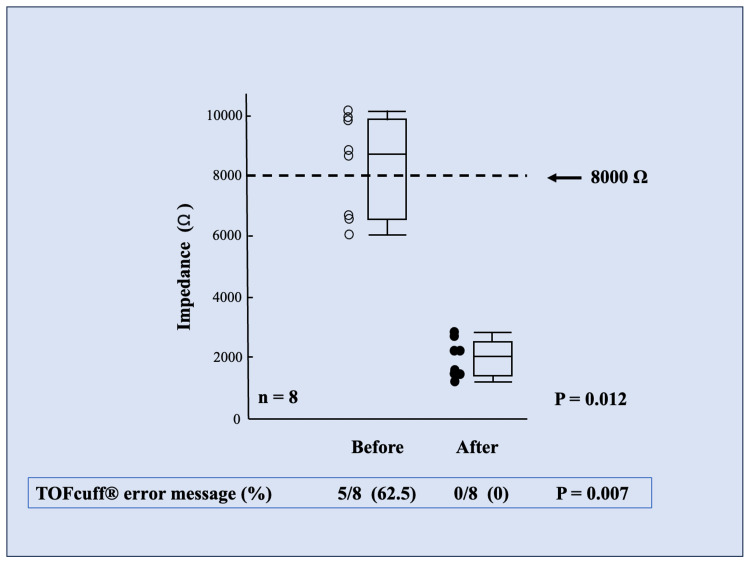
Electrical impedance between TOF-Cuff® electrodes and patients’ skin surface The collective data showing the electrical impedance between TOF-Cuff® electrodes and patients’ skin surface before and after the application of ECG cream on patients’ skin surface are shown. Please note the frequency of the TOF-Cuff® error message, which indicates unmeasurable TOF-Cuff® values, upon the measures did not note after the ECG cream application in surgical patients.

Table 2 presents the demographic data for the current study. The eight enrolled patients were > 70 years of age, with BMI values ranging from normal weight to overweight. ECG cream application between the patient’s skin surface and TOF-Cuff® electrodes decreased the skin-TOF-Cuff® electrode impedance (median (IQR) Ω) from 8,600 (6,450-9,775) to 2,000 (1,600-2,600) (P = 0.012) (Figure 4). Before the application of the ECG cream to the surgical patient, the frequency of the TOF-Cuff® error message, which indicates unmeasurable TOF-Cuff® values, was > 60%. In contrast, we did not observe an unmeasurable message after the application (Figure 4).

**Table 2:** Patients’ demographic data in clinical impedance evaluation Patients’ demographic data (n = 8) in the current study. Data are shown as median (interquartile range), or number (%). BMI: body mass index; ASA PS: American Society Anesthesiologists’ physical status.

Age (years)	75 (74.3 to 77.3)
Sex (F)	5 (62.5)
BMI (kg^-1^ m^2^)	24.0 (19.1 to 25.3)
Lower leg circumference (cm)	34.0 (30.8 to 35.8)
ASA PS 2	7 (87.5)
ASA PS 3	1 (12.5)

## Discussion

Plausible artifacts from the technical origin in electromyography monitoring include cable motion artifacts, transducer noise from the electrode displacement, high skin-electrode impedance, intrinsic noise from the electromyograph monitor, and biomedical devices such as a pacemaker [9]. Of these factors, we have examined whether adding some modification during TOF-Cuff® monitoring could decrease the skin-electrode impedance in the current in vitro study. The application of water, ECG cream, and ECG gel similarly reduced the impedance values toward an appropriate range of less than 5,000 Ω for electromyography monitoring [9]. These results suggest that wet conditions using materials can decrease the impedance level between the patient's leg skin and the TOF-Cuff® electrodes. In addition, every clinician engaged in anesthesia requires that the wet condition for the TOF-Cuff® measurement must be adequate for at least several hours during anesthesia and that the material producing the wet condition has to be gentle for the patient's skin. Therefore, we employed an ECG cream in our clinical study.

In the current study, the TOF-Cuff® unmeasurable message, which indicated a high impedance level above 8,000 Ω, was more than 60% before the ECG cream application between the patient's skin surface and TOF-Cuff® electrodes. These results agree with a recent study documenting a high measurement failure rate when using TOF-Cuff® on the lower leg caused by high impedance upon the measurement [8]. In contrast, we did not note any TOF-Cuff® unmeasurable messages after the ECG cream application. The clinical results, in addition to our in vitro study, suggest that wet conditions produced by the ECG cream can decrease the impedance level between the patient's leg skin and the TOF-Cuff® electrodes, at least in the population included in the current study. The skin water content decreases with age, indicating a higher skin impedance in older people [10]. The fact also explains the high failure rate of the TOF-Cuff® measurements before ECG cream application and the cream's effectiveness in aged patients in the current study. Therefore, our method employing the ECG cream upon TOF-Cuff® use in the lower leg via posterior tibial nerve stimulation is particularly appealing for elderly patients undergoing procedures such as arm/shoulder surgery, bilateral breast surgery, or axillary lymph node resections [6,7].

This study has several limitations. First, we did not determine the effects of materials other than water, ECG gel, and ECG cream on the skin-TOF-Cuff® electrode impedance. Thus, it is unclear whether other materials, including oil, decrease the impedance upon the TOF-Cuff® measurement. Second, we did not examine the role of the studied materials for a particular duration, such as several hours, during anesthesia. Therefore, the duration of the effect of the studied materials on skin-TOF-Cuff® electrode impedance remains unknown. Future studies are required to verify the clinically appropriate use of materials, including ECG cream, to enhance the secure TOF-Cuff® use in the lower leg, especially for elderly patients.

## Conclusions

In the current study, we first demonstrated that water, ECG gel, and ECG cream applied on the pseudo-skin between a model arm and TOF-Cuff® electrodes could reduce the impedance within the electrical circuit in an in vitro study. In addition, using ECG cream between the patient’s skin surface and the TOF-Cuff® electrodes decreased the skin-TOF-Cuff® electrode impedance to an appropriate level of less than 5,000 Ω in the clinical study.
